# Feasibility and Preliminary Efficacy of the Biya Yadha Gudjagang Yadha: Healthy Dads Healthy Mob Program

**DOI:** 10.1002/hpja.70078

**Published:** 2025-07-28

**Authors:** Jake C. MacDonald, Nathan Towney, Kathleen J. Butler, Myles D. Young, Lee M. Ashton, Briana L. Barclay, Philip J. Morgan

**Affiliations:** ^1^ Office of Indigenous Strategy and Leadership University of Newcastle Newcastle Australia; ^2^ Aboriginal and Torres Strait Islander Research Strategy and Leadership, Research and Innovation The University of Newcastle Newcastle Australia; ^3^ The School of Psychology The University of Newcastle Newcastle Australia; ^4^ Centre for Active Living and Learning University of Newcastle Newcastle Australia

**Keywords:** child health, codesign, indigenous health, men's health, parenting, yarning

## Abstract

**Issue Addressed:**

The important link between culture, health, and wellbeing is often overlooked when providing parenting support for Aboriginal fathers. This Aboriginal‐led, community co‐designed study was the first programme aimed to improve the health and wellbeing of Aboriginal fathers and their children living on Darkinjung Country (Central Coast NSW, Australia).

**Methods:**

Single arm, pre‐post feasibility trial including qualitative (yarning) and quantitative (survey & anthropometry) measures assessing a 9‐week health and wellbeing programme tailored for Aboriginal fathers and their primary school aged (5–12 years) children living on Darkinjung Country.

**Results:**

Feasibility was achieved with nearly all a priori benchmarks met; fidelity 93% (benchmark ≥ 80%), attendance 79% (benchmark ≥ 70%), home‐activity compliance 93% (benchmark ≥ 60%), retention 86% (benchmark ≥ 70%), satisfaction 5/5 (benchmark = 4/5). Recruitment capability (7 families, 15 participants) was not achieved (benchmark: 20 families). Regarding preliminary efficacy, large effect sizes (*d* ≥ 0.8) were evident for most assessed outcomes in both fathers and children. Qualitative findings indicate that Aboriginal fathers living on Darkinjung Country find the programme to be acceptable.

**Conclusions:**

Program feasibility was confirmed with high levels of program attendance, retention, and participant satisfaction. Large effect sizes were supported by very positive qualitative feedback from participants. Future research involving Aboriginal fathers should consider these findings in the development of culturally responsive parenting support.

**So What:**

This new health and wellbeing programme designed for Aboriginal fathers and their children achieved programme feasibility outcomes and reports promising qualitative and quantitative findings. This research could be used to inform future development of parenting programmes involving Aboriginal fathers and their children.

**Trial Registration:** Clinical Trials registry: ACTRN12623000901606

## 1 Introduction

The social positioning of Indigenous peoples globally is a consequence of the underdevelopment of their communities as an objective of the colonial project [1]. The ongoing effects of colonisation continue to negatively impact the health and wellbeing of Aboriginal people in Australia [2]. Significant disparities in morbidity and mortality rates have been reported between Aboriginal and non‐Aboriginal groups [3], with cardiovascular disease being the leading cause of death of Aboriginal people [4]. Whilst the health of Aboriginal people is well reported, it is critical to acknowledge that nearly half of reported illnesses are due to potentially modifiable risk factors [5], and the interconnected relationship between culture, health, and wellbeing is not adequately understood in the provision of care of Aboriginal people [6].

Although Aboriginal parents and their children have been identified as a health priority group [4], culturally relevant health and wellbeing support is nearly non‐existent, particularly for Aboriginal fathers [7]. This is concerning given that Aboriginal men are nearly three times more likely to develop cardiovascular disease than non‐Aboriginal men [4], have an average life expectancy that is a decade shorter than non‐Aboriginal men [8], and have the worst overall health and wellbeing outcomes of any group in Australia [4]. In addition, Aboriginal men have had their important familial roles disrupted through the forced removal of children, dispossession of Country, and the generational impacts of disconnection from family and community [2] which has resulted in Aboriginal men experiencing feelings of meaninglessness, alienation, and loss of culture [9].

Our recent scoping review of randomised trials testing parenting interventions in Australia [10] determined that Aboriginal fathers were significantly under‐represented and very few researchers consulted with Aboriginal communities when designing programmes. To address this knowledge gap, our research team undertook an extensive codesign process with the Darkinjung community on the Central Coast of New South Wales, Australia to develop a new programme for Aboriginal fathers and their children; biya yadha gudjagang yadha: Healthy Dads Healthy Mob. Previous formative research has been published with the aim to understand the roles, experiences, and needs of Aboriginal fathers living on Darkinjung Country [11], and a cultural adaptation process to design the current study has also been reported [12].

This Aboriginal‐led study privileged the voices of Aboriginal fathers, had cultural governance from a self‐determining cultural affirmation panel, and was codesigned with Aboriginal community members living on Darkinjung Country to create a strengths‐based health and wellbeing programme for Aboriginal fathers and their children. The biya yadha gudjagang yadha: Healthy Dads Healthy Mob programme included some elements of the original Healthy Dads Healthy Kids (HDHK) programme, which has previously been delivered to non‐Aboriginal families, and has reported a positive impact on child physical activity levels [13], and a reduction in bodyweight in fathers [14]. This paper aims to assess the feasibility and preliminary efficacy of the biya yadha gudjagang yadha: Healthy Dads Healthy Mob programme.

## 2 Methods

### 2.1 Governance

Aboriginal people have the right to participate in decision‐making in matters which impact their health and wellbeing [15], and involving Aboriginal people in the development of health research increases study relevance [16]. The biya yadha gudjagang yadha: Healthy Dads Healthy Mob program was codesigned in partnership with a self‐determining cultural governance group self‐defined as the cultural affirmation panel. The cultural affirmation panel had oversight of all elements of the research including the design of study methods, program adaptation, and program implementation.

The cultural affirmation panel consisted of eight stakeholders representing the local Darkinjung community, the University of Newcastle, and Eleanor Duncan Aboriginal Services. All members of the cultural affirmation panel were Aboriginal, no panel members identified as Torres Strait Islander. Some cultural affirmation panel members were Elders, and there was both male and female representation.

### 2.2 Research Team

It is important to identify the relationality of our research team as a required element of Indigenous research practice [17]. Authors include Aboriginal researchers JM, NT & KB, along with non‐Aboriginal researchers MD, LA, BB & PM. Further information regarding the cultural identity of the research team can be found in Appendix 1.

### 2.3 Program Information

Biya yadha gudjagang yadha: Healthy Dads Healthy Mob was a healthy lifestyle programme specifically designed for Aboriginal fathers and their primary‐school aged children. The study was facilitated by Aboriginal men and developed from an extensive community codesign process to adapt the original HDHK programme to be culturally relevant for Aboriginal fathers and their children living on Darkinjung Country [11, 12]. Participants took part in an education and practical session, followed by a healthy shared meal once a week over a 9‐week period. The education sessions were themed, with a new culturally relevant health‐related topic covered each week. The practical sessions had three components: rough‐and‐tumble play, sport skills, and fitness. The end‐of‐session shared meal was an opportunity for families to develop relationships and practise healthy eating strategies discussed in the programme. The weekly sessions occurred over a 90‐min period, with the first week being a dads‐only session where participating fathers were given an overview of the programme. All other sessions throughout the programme were completed together by fathers and their children. A take‐home handbook with fun physical activities and information aligned with topics discussed in each week of the programme was provided to families to be completed at home. The programme commenced on 12th October 2023, and the final session took place on 7th December 2023.

### 2.4 Participant Eligibility

Participants were eligible for the study if they met each of the following criteria:

- Identify as Aboriginal and/or Torres Strait Islander.
- Live on Darkinjung Country.
- Are a father or father‐figure to a child aged 5–12 years.
- Pass a pre‐exercise health‐screening questionnaire and/or provide GP clearance to participate.

Note: All children were eligible to participate if they were attending primary school in 2023 (Aged 5–12 years).

### 2.5 Recruitment

An information brochure was developed which included an overview of the programme, a QR code to express interest in participating, and an email address to request further information. Brochures were distributed using two strategies:

1. Brochures distributed by Eleanor Duncan Aboriginal Services.
2. Brochures distributed through local primary schools on the Central Coast of NSW.

### 2.6 Study Setting

The cultural affirmation panel advised that the appropriate location to deliver the programme was on site at Eleanor Duncan Aboriginal Services, which is the sole Aboriginal community‐controlled health service on the Central Coast, has appropriate facilities, and was considered to be a culturally safe location for Aboriginal families.

### 2.7 Measures

Culturally appropriate evaluation methods are required to understand the impact of research involving Aboriginal people [18]. The cultural affirmation panel advised that both quantitative and qualitative methods were required to determine the feasibility of the program. The research team worked with the cultural affirmation panel to codesign the following measures.

### 2.8 Quantitative Measures

Quantitative assessments took place at baseline (pre‐program) (5th October 2023) and end of program (7th December 2023). Refer to Table S1 for a full description of quantitative measures. Briefly, the a priori established primary outcomes for the study were achievement of feasibility targets for recruitment (20 families), fidelity (≥ 80%), attendance (≥ 70%), compliance (≥ 60%), retention (≥ 70%), and program satisfaction (4/5). The a priori targets for these feasibility outcomes were registered with the Australia and New Zealand Clinical Trials Registry prior to starting the study and were based on previous feasibility trials of family‐based interventions [19, 20, 21]. Program fidelity was measured by observation, compliance was measured by a post‐program handbook review by the facilitator, and satisfaction was measured by survey. Secondary outcomes were assessed via survey questionnaire to measure preliminary efficacy, which included cultural identity, physical activity, and nutrition. Anthropometry measures included objectively measured weight and BMI, which were assessed at Elenor Duncan Aboriginal Services. Demographic information included participant age, Aboriginal or Torres Strait Islander status, relationship status, relationship to participating child, and country of birth. Age and sex of the enrolled child were also collected.

### 2.9 Qualitative Measures

Yarning was employed as a culturally grounded qualitative research method to explore programme feasibility and preliminary efficacy. As an Indigenous research methodology, yarning facilitates an open and relational approach to data collection that fosters cultural safety and trust amongst participants [22].

A post‐program yarning session was conducted on 14 December 2023. This session was convened by the program facilitator JM and included participating fathers. To acknowledge their contributions, each participant was presented with a $30 AUD gift voucher. The session began with social yarning over a shared meal, followed by research topic yarning conducted in a single 2‐h group discussion.

A semi‐structured guide codesigned with the cultural affirmation panel was used. Questions included:

- What worked well for you during the program?
- What could we do better in the programme?
- Has there been any change in your life or your child's life as a result of the program?

### 2.10 Data Collection and Analysis

Data were collected and managed using REDCap electronic data capture tools hosted at the Hunter Medical Research Institute, which is a partner to the University of Newcastle [23, 24]. Descriptive analyses (i.e., percentage and frequency counts) were conducted to assess recruitment, fidelity, attendance, compliance, retention, and program satisfaction. For preliminary intervention efficacy measures, a paired *t*‐test was used to compare mean scores at pre‐intervention and post‐intervention, and effect sizes calculated using Cohen's *d*. Effect sizes were interpreted as small (*d* = 0.2), medium (*d* = 0.5) or large (*d* = 0.8) [25]. Yarning data were collected using audio recording and field notes and analysed by one Aboriginal researcher JM without the use of coding software. This method was guided by previous qualitative research conducted with Aboriginal and Torres Strait Islander men [26]. The process adhered to the six stages of employing thematic networks [27] and is informed by the positionality of the single analyst who is an Aboriginal father from the Darkinjung community.

### 2.11 Participant Privacy Protection

The cultural affirmation panel requested that the identity of program participants be protected. Any additional information not reported in this paper regarding the study participants is anonymous.

### 2.12 Ethics

This research was approved by the Aboriginal Health & Medical Research Council of NSW's Ethics Committee, Application ID: 32414513, and registered with the University of Newcastle Human Research Ethics Committee (H‐2023‐0137), and the Australian New Zealand Clinical Trials Registry (ACTRN12623000901606). This research is in alignment with the consolidated criteria for strengthening reporting of health research involving Indigenous peoples.

## 3 Results

**FIGURE 1:**
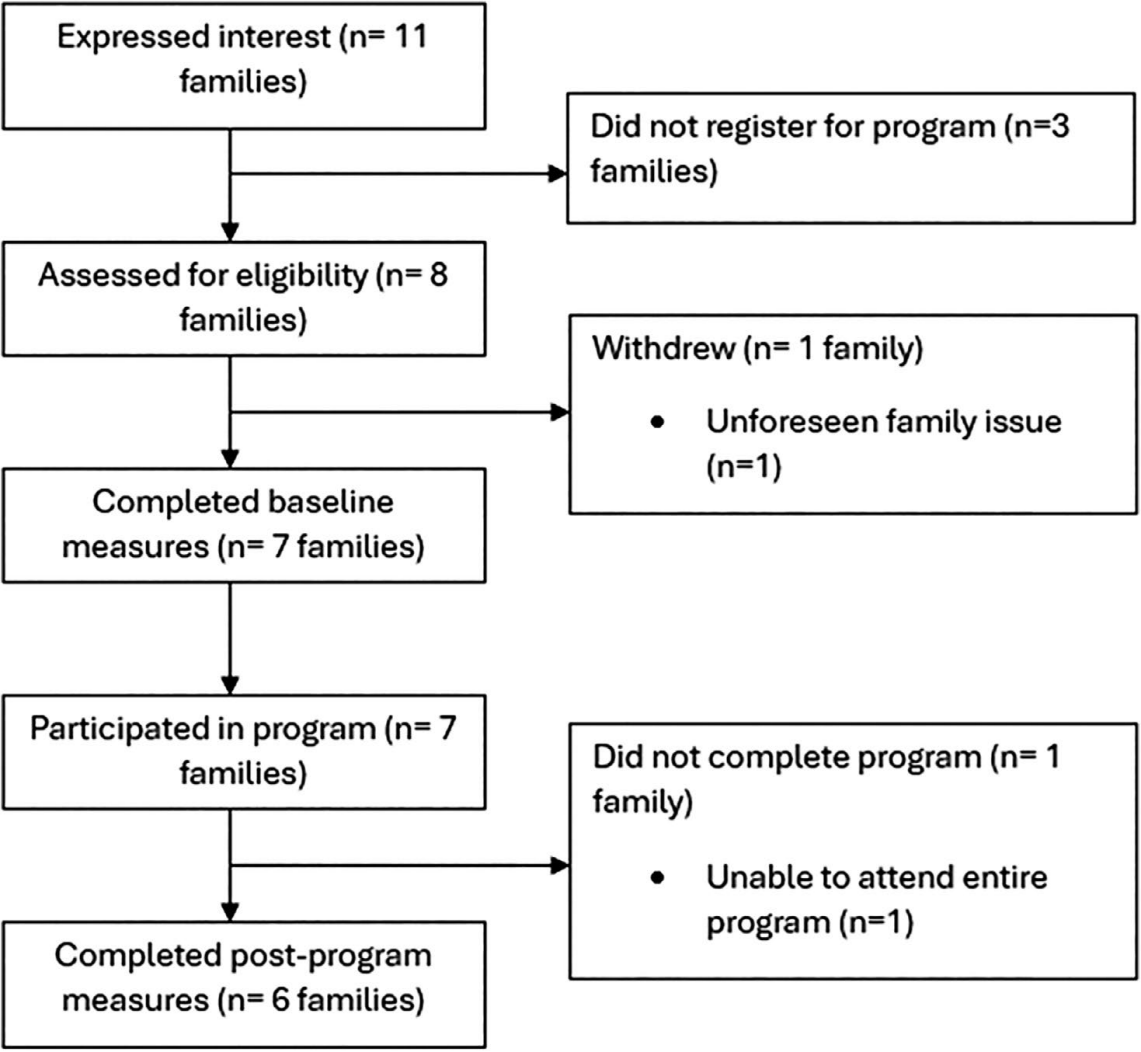
Flow of study participants.

The flow of participants involved in the study is shown in Figure 1, while characteristics of study participants at baseline can be found in Table S2.

### 3.1 Quantitative Results (Primary Outcomes)

A full description of feasibility outcomes can be found in Table S3. Briefly, a priori benchmarks were met for most feasibility outcomes including fidelity 93% (benchmark ≥ 80%), attendance 79% (benchmark ≥ 70%), compliance with home‐based programme 93% (benchmark ≥ 60%), retention 86% (benchmark ≥ 70%), overall mean programme satisfaction score 5/5 (benchmark = 4/5), while recruitment capability (7 families) fell short of benchmark (20 families).

### 3.2 Quantitative Preliminary Efficacy Findings

A full description of secondary outcomes for preliminary efficacy can be found in Tables S4 and S5. Briefly, large effect sizes were achieved representing increases in father cultural identity (*d* = 1.10, *p* < 0.05), days per week meeting physical activity recommendations (+2.3 days/week, *d* = 1.17, *p* < 0.05), and overall dietary intake score (*d* = 1.64, *p* < 0.05). In children, there were large effect sizes observed for days per week meeting physical activity recommendations (+3.2 days/week, *d* = 2.71, *p* < 0.01), and overall dietary intake score (*d* = 1.99, *p* < 0.01). Small effect sizes were observed for the anthropometry outcomes in both fathers and children.

### 3.3 Qualitative Results

Key findings from the post‐program yarn are presented in Table 1. Briefly, study participants spoke about the positive elements of the program, discussed how the program could be improved, and the program's impact. Notably, participants provided overwhelmingly positive feedback on the improvement of the physical, social, and emotional health of both fathers and children as a result of the program.

**TABLE 1:** Post‐program yarn findings.

Question asked	Theme	Illustrative quote
*What worked well for you during the program?*	Quality time with children	*“I don't normally get time when you're just playing around and doing some structured stuff, I found that to be really beneficial, and I've probably got a better relationship with [name of child] now through that.”*
	Dads and kids connecting through culture	*“That was pretty cool I thought, like all blackfellas coming together, and just connecting through culture as well, like a lot of the boys wanted to kind of talk about that stuff, and I don't think you get as many opportunities to connect through culture with your kids as well.”*
	Kids seeing dads being role models	*“I like seeing other dads be role models for their kids, that's a good thing for kids to learn young, about committing to other people. If you say you're going to do something, you've got to do it, it is about seeing other dads show those behaviours. I think the kids pick up on that, whether they realise it or not.”*
	Connecting with other Aboriginal fathers	*“I think a big thing for me was that sense of community as well, like having fellas that feel like we are connected in non‐family ways, but just as fellas who have kids that are similar ages and you're trying to look after them, I think that's pretty cool.”*
	The post program meal was a valuable addition to the program	*“The feed after the program was good, it was good healthy food too. Like I found, jeez that stuff we had the other night was pretty good, I wish we could have that tonight.”*
*What could we do better in the program?*	An option to condense the 9‐week program into a weekend camp	*“It's a big time commitment to go for 9 weeks straight, to show up for a couple of hours. You could do like a weekend version, where you go to like a camp or something, and you stay there for a few days and kind of compress it.”*
	Include cultural dance as a physical activity	*“I reckon you could involve dance, because there were girls there, and I mean boys could do dance too, they need to learn that culture. That ties in story, and story around dance, because art and story, dance, they all go sort of hand in hand.”*
	When in other communities include knowledge holders to teach local stories where possible	*“If you think about other communities, you can bring in people with cultural knowledge to teach their story for their community.”*
*Has there been any changes in your life or your child's life as a result of the program?*	Continued increased physical activity levels	*“I hadn't been to a gym in years, and I liked the idea of running around every Thursday. I was sore the first week, I haven't really moved much. I found every week I was getting better and better, and I was losing weight. And at the end of it, I decided I wanted to keep going with this, so I ended up going to the gym, and I'm going to continue going.”*
	Continued use of healthy diet strategies in the family	*“I've probably been making better choices as far as what I eat, simply because I enjoyed the stuff we were eating there. That greasy stuff, I'm not real wrapped in that now after eating healthy.”*
	Improved relationship between fathers and children	*“I can see a change in him for sure, and in our relationship, for the better, definitely for the positive.”*
	Stronger sense of community amongst fathers	*“That's the strength of blackfellas sticking together, we all know each other, we know the kids, and it's like the old days, there's more community. Like where I come from, there was a community that cared for each other, people knew who you were, they knew who your parents were, and if you got into strife, they knew where to come calling. It has been good here with the fellas, it feels like that again.”*

## 4 Discussion

The aim of this paper was to report the feasibility and preliminary efficacy of the biya yadha gudjagang yadha: Healthy Dads Healthy Mob program. Overall, feasibility was achieved with targets met for retention, fidelity, attendance, compliance, and program satisfaction. There were also large effect sizes for indicators of preliminary efficacy including cultural identity, physical activity, and diet. Participating fathers also provided overwhelmingly positive feedback supporting program acceptability.

A promising aspect of the study was that almost all feasibility targets were exceeded, which indicates that the biya yadha gudjagang yadha: Healthy Dads Healthy Mob program was feasible. Attendance targets were achieved with an average attendance rate of 79% across the span of the program. The achievement of the attendance target is encouraging, particularly since Aboriginal fathers have been underrepresented in previous parenting trials conducted in Australia [10]. The take‐home handbook activity completion compliance target was achieved along with the fidelity target, which indicates the program facilitator effectively delivered the program content. Delivering the program with high fidelity is particularly important given that all elements of the program were codesigned to be culturally relevant for Aboriginal fathers and their children living on Darkinjung Country. Program acceptability is supported by retention targets being met with 86% of participants retained, which is complemented by very positive program satisfaction scores where all targets were met. Of note, maximum scores (5/5) for program enjoyability and overall program rating were achieved. The positive satisfaction scores were supported by qualitative feedback that fathers felt they had an improved relationship with their children as a result of the program. However, it is important to note that the recruitment target was not met, with only seven families participating. This may be explained due to a delay in ethics approvals where the window for recruitment was less than a month, which likely impacted the total number of families that enrolled. While the overall numbers for recruitment were low, the recruitment rate is comparable to a previous program conducted by the authors of this study, which targeted fathers and had a longer recruitment period [19]. With a larger window for recruitment, the authors are confident that the recruitment target of this study would be met.

The biya yadha gudjagang yadha: Healthy Dads Healthy Mob program had a positive impact on self‐reported physical health measures for both fathers and children. Large effect sizes were observed in days per week meeting physical activity recommendations for fathers and children, and large effect sizes for dietary intake score for fathers and children. Whilst there appears to be a large effect on father and child self‐reported physical health behaviours, this information should be tempered with small effect sizes observed for both father and child BMI. However, in the yarn, fathers did report continued increased physical activity levels and continued healthy eating habits post‐program. These findings are significant given that cardiovascular disease is the leading cause of death of Aboriginal men in Australia [4]. We believe the positive outcomes discussed here were influenced by this study giving attention to the social, emotional, and cultural factors that influence the physical health of Aboriginal men. We advise that those providing parenting support for Aboriginal men do not address physical health in isolation without consideration of these factors.

For Aboriginal people, there is an interconnected relationship between culture and social and emotional wellbeing [28]. After participating in the biya yadha gudjagang yadha: Healthy Dads Healthy Mob program, a large effect on fathers' cultural identity was observed. This was supported by qualitative findings indicating the program had a positive impact on fathers' social and emotional health. Whilst qualitative findings highlighted that participants found this program to be an opportunity for fathers to connect with their children through culture, there remains very little research on what Aboriginal fathers would like to gain from a parenting program. A recent study [29] reviewed data from the Longitudinal Study of Indigenous Children (LSIC), which asked Aboriginal male parents what supports they require in fulfilling their role as a father. Findings highlighted the importance of parenting support factoring cultural elements which impact on Aboriginal men's health and wellbeing, with authors calling for parenting support to move away from a deficit approach to caring for Aboriginal men and take a strengths‐based approach. In this context, we believe that future parenting research involving Aboriginal fathers must be reoriented to consider the cultural values, knowledges, and lived experiences of Aboriginal men as a source of strength, and that programs designed for Aboriginal families be delivered by Aboriginal people.

A recent study by Canuto and Gaweda [30] reviewed programs designed for Aboriginal and Torres Strait Islander men and described key study characteristics. Authors of the review identified inconsistency amongst program elements, which included origin of design, governance, facilitators, funding, structure, outcomes, and evaluation. Of all 54 studies included in the review, only six had a focus on fathers, with a high level of variation across program design elements. Review findings highlight a lack of parenting programs for Aboriginal and Torres Strait Islander men, inconsistency across the field of research, and the need for a considered approach to address the health and wellbeing of Aboriginal and Torres Strait Islander fathers. The biya yadha gudjagang yadha: Healthy Dads Healthy Mob program took an innovative approach to address a gap in the field of Aboriginal men's health and wellbeing research and was the first health and wellbeing program designed specifically for Aboriginal fathers and their primary‐school aged children living on Darkinjung Country. This study is an example of an Aboriginal‐led community codesigned program that could be used to inform the development of future parenting support.

## 5 Strengths and Limitations

This study had several strengths. First, the study tested a program codesigned with members of the Darkinjung community. The research was also governed by a self‐determining cultural affirmation panel. Study strengths also include Aboriginal research leadership and the program being facilitated by a local Aboriginal father who is part of the Darkinjung community, and the use of both quantitative and qualitative measures. Social desirability bias may have been a potential limitation as the relationships developed throughout the program may have influenced participants self‐report measures. However, we also acknowledge that the relationships between the researcher and participants in an Aboriginal community context could be a potential strength as cultural safety may increase the likelihood of honest self‐reporting. We acknowledge other limitations. First, this study had a small sample size, and while the study results are promising, findings should be interpreted with caution due to the limited number of participants. There was a small window for recruitment due to a longer than expected delay in ethics approval which likely impacted the total enrolment of families into the program. Participants were required to gain medical clearance from a GP if they were unable to pass a pre‐enrolment health screening which may be a potential study limitation. Furthermore, the Central Coast of NSW is a geographically large region; however, this program was conducted at only one location which may have impacted access for potential study participants.

## 6 Conclusion

The biya yadha gudjagang yadha: Healthy Dads Healthy Mob program was the first health and wellbeing program designed for Aboriginal fathers and their primary‐school aged children living on Darkinjung Country. The aim of this paper was to report the feasibility trial results of the program. The feasibility of the study was supported by high levels of program attendance, retention, and participant satisfaction. These findings are complemented by very positive qualitative feedback indicating that Aboriginal fathers living on Darkinjung Country find the program to be acceptable. Based on the findings of this study, we recommend that the positive indicators of preliminary efficacy are further investigated in a larger trial.

## Ethics Statement

This research was approved by the Aboriginal Health & Medical Research Council of NSW's Ethics Committee, Application ID: 32414513, and registered with the University of Newcastle Human Research Ethics Committee (H‐2023‐0137), and the Australian New Zealand Clinical Trials Registry (ACTRN12623000901606).

## Conflicts of Interest

The authors declare no conflicts of interest.

## Supporting information


Table S1.

## Data Availability

The de‐identified data we analysed are not publicly available, but requests to the corresponding author for the data will be considered on a case‐by‐case basis in conjunction with the cultural governance of this project.

## Appendix 1 Author Positionality Statements

1. Jake MacDonald is a Ngarabal man with family ties to Emmaville and Deepwater, Northern NSW, Australia. Jake has lived on Darkinjung Country, the Central Coast of NSW his entire life, and is part of the Darkinjung community. Jake is a father, educator, and PhD student at the University of Newcastle. Jake has a background in teaching, previously working as an Aboriginal Education consultant.
2. Nathan Towney is a Wiradjuri man from Wellington NSW. Nathan is a father to Archie and Charlotte. He also has a background in teaching, spending over 20 years with the NSW Department of Education in a variety of roles. Nathan is currently the Pro Vice‐Chancellor Indigenous Strategy and Leadership and the Head of the Wollotuka Institute at the University of Newcastle.
3. Kathleen Butler belongs to the Worimi and Bundjalung peoples of coastal New South Wales. Living on Darkinjung Country, Kath is a mother of three. She is currently the Director, Indigenous Research Strategy and Leadership in the Research and Innovation Division of the University of Newcastle. Kath has held numerous academic roles in the past 25 years at the University of Newcastle and passionately believes in the power of education.
4. Myles Young is a non‐Indigenous man, father, and academic in clinical and health psychology at the University of Newcastle. He has a particular interest in working with research end‐users to design and test new ways of improving men's physical and mental health.
5. Lee Ashton is a non‐Indigenous man and a father to two children. He has a background in men's health and family‐based health programs at the University of Newcastle.
6. Briana Barclay is a non‐Indigenous woman who lives on Darkinjung Country, the Central Coast of NSW. She has professional experience in the field of nutrition and dietetics and family‐based health programs at the University of Newcastle.
7. Philip Morgan is a non‐Indigenous man and a father to three girls. He has a background in physical education and expertise in the design and evaluation of programs for fathers and men.
