# Supplementary material for: Feasibility and Preliminary Efficacy of the Biya Yadha Gudjagang Yadha: Healthy Dads Healthy Mob Program
Source: Health Promot J Austr. 2025 Jul 28;36(4):e70078. doi: 10.1002/hpja.70078 (PMC12301869; doi:10.1002/hpja.70078)
Supplement: Supplementary file 1 — Table S1. [file HPJA-36-0-s001.docx]

Appendix 2.

**Supplementary Table S1:** Overview of primary and secondary quantitative outcome measures

| **Measure** | **Description** | | **Timepoint** | |
| --- | --- | --- | --- | --- |
| ***Primary outcome*** | | | | |
| *1. Recruitment capability* | - **Measurement tool:** Audit of study enrolment logs. - **Indicator of success:** achievement of recruitment targets for participants (recruitment of 20 families). | | Baseline | |
| *2. Fidelity of program delivery* | - **Measurement tool**: Direct observation of program facilitator by a member of the research team during ≥two sessions. A fidelity checklist was developed for the purpose of the study to ensure all content was delivered as intended. To support this, weekly post session reflections were completed by the facilitator. - **Metrics/questions**: Observers ‘ticked’ checklist for successful delivery of session content for education and practical activities. For education sessions this is reported as % of PowerPoint slides successfully delivered in relation to total number of slides intended to be delivered. For practical, this is % of activities successfully delivered in relation to total number of activities intended to be delivered. For the reflections, the facilitator was asked to indicate any sessions where they were unable to deliver as intended with option to elaborate where necessary. - **Completed by:** direct observations completed by member of research team, reflections completed by facilitator. - **Indicator of success**: Successful delivery of at least 80% of the session content by facilitator. | | Post-program | |
| *3. Attendance* | - **Measurement tool**: assessed using workshop attendance checklists at the father-only workshops and the 8-eight x father-child sessions. - **Metrics/questions**: Reported as % attendance at the fathers-only workshop and % attendance for father-and-child sessions on average across the eight weeks. - **Completed by:** program facilitators - **Indicator of success:** at least 70% average attendance at the fathers-only workshop and the father-child sessions on average across the eight weeks. | | Post-program | |
| *4. Compliance with home-based program* | - **Measurement tool:** assessed by collecting home-program handbooks at the end of the last session and recording the number of home tasks completed by the fathers and their child. - **Completed by:** member of research team. - **Indicator of success:** Successful compliance will be defined as Families completing an average of at least 60% of the home-based tasks in the activity handbook. | | Post-program | |
| *5. Retention* | - **Measurement tool:** Number of participants completing post-program measures. - **Indicator of success:** at least 70% retained at end of the program and completed post-program measures. | | Post-program | |
| *6. Program satisfaction* | - **Measurement tool:** assessed using post-program process evaluation online survey developed for the purpose of the study. - **Metrics/questions:** questions focused on participants’ satisfaction with overall program and program facilitator (e.g., *Overall, I enjoyed the Healthy Dads, Healthy Mob: biya yadha gudjagang yadha program*). Responses were on a 5-point Likert scale where strongly disagree = 1 and strongly agree = 5. - **Completed by:** fathers - **Indicator of success:** defined as a mean score of at least 4 out of 5 for satisfaction items measured via questionnaire using a 5-point Likert scale. | | Post-program | |
| ***Secondary outcomes – preliminary efficacy*** | | | | |
| *Culture and Identity* | - **Measurement tool:** 3-item cultural engagement scale [31]. - **Metrics/questions**: 3 questions asked including *How often do you participate in Aboriginal/Torres Strait Islander activities and events? Do you feel connected to your homeland or traditional country? Do you feel connected to your culture?* Responses were on a scale from 0-4 including Never = 0, Rarely =1, Sometimes = 2, Often =3, and Always = 4. A total score was created combining all 3-items. - **Completed by:** Fathers. | | Baseline, Post-program | |
| *Father’s Self Report MVPA (mins/week)* | - **Measurement tool:** Adapted version of the Godin Leisure Time Exercise Questionnaire [32]. - **Metrics/questions:** Fathers reported average weekly bouts of moderate and vigorous physical activity and average bout length [33]. Values in each category were multiplied and summed to give an overall measure of weekly MVPA. - **Completed by:** Fathers. | | Baseline, Post-program | |
| *Father-child co-physical activity (days/week)* | - **Measurement tool**: 2-items adapted from the Youth Media Campaign Longitudinal Survey [34]. - **Metrics/questions:** Fathers reported on days per week they were physically active with their child one-on-one and with one or more family member. - **Completed by:** Fathers. | | Baseline, Post-program | |
| *Dietary Intake* | - **Measurement tool:** Adapted version of the Many Rivers Short Food Frequency Questionnaire (MRSFFQ) [35]. - **Metrics/questions:** dietary Intake is assessed on ordinal scales for 10 dietary outcomes. Fathers responded about themselves and on behalf of their children. Intake of healthy, nutrient-dense core food items of fruit, vegetables and water was scored on a scale 0 – 4 for fruit and 0 – 5 for vegetables and water with higher numbers corresponding to higher numbers of serves a day. Energy-dense, nutrient poor food items of hot chips, salty snacks, confectionary, sweet foods, fast food, fruit juice and sweetened beverages were reverse scored on a scale of 0 – 6 with higher numbers corresponding to lower numbers of serves per week. Composite variables were then formed through summing across healthy, nutrient-dense core foods, Energy-dense, nutrient poor food and all items. - **Completed by:** Fathers. | | Baseline, Post-program | |
| *Weight (kg)* | - Objectively measured using calibrated electronic scales. Measured in regular clothing, without shoes on a digital scale to 0.01 kg (model CH-150kp, A&D Mercury Pty Ltd, Australia) - Weight was recorded at least twice until two measures fell within a range of 0.1 kg, averaged for the analysis. | | Baseline, Post-program | |
| *BMI (fathers)* | - Calculated using the standard formula weight (kg)/height in m^2^ | | Baseline, Post-program | |
| *BMI Z-score (Children)* | - Children’s BMI-z scores were calculated using age- and sex-adjusted standardized scores (z-scores) based upon the UK reference data and LMS methods [36]. - International Obesity Task Force cut points were used to determine overweight or obesity [37]. | | Baseline, Post-program | |
| **Supplementary Table S2**: Characteristics of study participants at baseline. | | | |  |
| **Characteristic** | **Fathers (n = 7)** | | |  |
|  | **Mean** | **SD** | |  |
| Age (y) | 42.09 | 11.71 | |  |
|  | **n** | **%** | |  |
| Relationship to participating child |  |  | |  |
| *Biological Father* | 6 | 87.50% | |  |
| *Grandfather* | 1 | 12.50% | |  |
| Born in Australia | 7 | 100% | |  |
| Identifies as Aboriginal Australian | 7 | 100% | |  |
| Relationship status |  |  | |  |
| *Married* | 5 | 71.43% | |  |
| *Living with partner* | 1 | 14.29% | |  |
| *Separated* | 1 | 14.29% | |  |
|  | **Children (n = 8)** | | |  |
|  | **Mean** | **SD** | |  |
| Age (y) | 7.96 | 2.51 | |  |
|  | **n** | **%** | |  |
| Sex |  |  | |  |
| *Male* | 6 | 75% | |  |
| *Female* | 2 | 25% | |  |

**Primary outcomes**

**Supplementary Table S3:** Overview of feasibility results (primary outcome).

| **Feasibility outcome** | **a-priori feasibility benchmark** | **Met benchmark?** | **Key results** |
| --- | --- | --- | --- |
| *Recruitment capability* | Recruitment of 20 families. | **🗴** | - Total of 7 families enrolled. - Most fathers enrolled one child (n=6, 86%), while one father (14%) enrolled two children. |
| *Fidelity* | Delivery of ≥80% of the session content by facilitators during the fathers-only workshops and the dads & children education and practical sessions | **🗸** | - For the father-only workshop, 96% of education session was successfully delivered. - For the dads and kids session 100% of the education session content and 82% of practical session content was successfully delivered as intended. |
| *Attendance* | At least 70% attendance at the fathers-only workshops and the father-and-child sessions on average across the eight weeks | **🗸** | - 71% (n=5) of fathers attended the dads-only session at the start of the program. - Average attendance for the eight-weekly father–child sessions was 78.6%. |
| *Compliance with home tasks* | Complete at least 60% of the home-based tasks in the activity handbook. | **🗸** | - The average proportion of families that completed the minimum requirement for weekly home-based tasks (at least 1 activator and 1 sport skill activity) was 93%. |
| *Retention* | At least 70% retained at end of the program | **🗸** | - One father dropped out of the program due to family circumstances leaving 86% (n=6 families) retained at the end of the program for post-program measures. |
| *Program satisfaction* | Mean score of ≥4 out of 5 for satisfaction items measured via questionnaire using a 5-point Likert scale. | **🗸** | - On a scale of 1 (poor) to 5 (excellent), the mean (standard deviation (SD)) overall program satisfaction score was 5.0 (0.0). Likewise overall facilitator rating was 5.0 (0.0). - On a scale of 1 (strongly disagree) to 5 (strongly agree), fathers rated the dads and kids’ sessions highly with maximum scores received for the education sessions (mean: 5.0, SD:0.0) and practical sessions (mean: 5.0, SD:0.0), while the dads-only session rated slightly lower (mean 4.7, SD: 0.5). |

**Supplementary Table S4:** Changes in outcomes between baseline and post-program (9-weeks) for fathers

| **Outcome** | **Baseline, mean (SD)** | **Post-intervention mean (SD)** | **Mean diff (SD)** | **95%CI** | ***t (df)*** | **Cohen *d*** |
| --- | --- | --- | --- | --- | --- | --- |
| Culture and Identity |  |  |  |  |  |  |
| *Participation in Aboriginal/Torres Strait Islander activities and events* | 3.0 (1.1) | 4.0 (0.0) | 1.0 (1.1) | -0.1, 2.1 | *2.2 (5)* | 0.91 |
| *Connected to homeland or traditional country* | 2.8 (1.0) | 4.0 (0.0) | 1.2 (1.0) | 0.1, 2.2 | ***2.9 (5)**** | 1.19 |
| *Connected to culture* | 3.5 (0.3) | 4.0 (0.0) | 0.5 (0.8) | -0.4, 1.4 | *1.5 (5)* | 0.60 |
| *Total culture and identity score* | 9.3 (2.4) | 12.0 (0.0) | 2.7 (2.4) | 5.2, 0.1 | ***2.7 (5)**** | 1.10 |
| Physical Activity |  |  |  |  |  |  |
| *Days/week meeting PA recommendations* | 3 (1.8) | 5.3 (1.4) | 2.3 (2.0) | 4.4, 0.3 | ***2.9 (5)**** | 1.17 |
| *MVPA (mins/week)* | 290.8 (233.8) | 502.5 (307.7) | 211.7 (343.7) | -149.0, 572.4 | *1.5 (5)* | 0.62 |
| Co-physical activity |  |  |  |  |  |  |
| *With child & family (days/week)* | 2.0 (1.7) | 4.0 (1.3) | 2.0 (2.2) | -0.3, 4.3 | *2.2 (5)* | 0.91 |
| *With child only (days/week)* | 1.8 (1.6) | 4.0 (1.3) | 2.2 (1.6) | -0.3, 4.6 | *2.3 (5)* | 0.94 |
| Anthropometry |  |  |  |  |  |  |
| *Weight (kg)* | 89.3 (16.3) | 89.1 (16.3) | -0.3 (1.6) | -1.9, 1.3 | *-0.4 (5)* | 0.18 |
| *BMI (kg/m^2^)* | 27.3 (3.9) | 27.2 | -0.1 (0.5) | -0.6, 0.4 | *-0.5 (5)* | 0.21 |
| Dietary intake |  |  |  |  |  |  |
| *Energy-dense, nutrient poor foods (range 0-42)* | 25.7 (7.4) | 35.2 (3.8) | 9.5 (8.3) | 0.8, 18.2 | ***2.8 (5)**** | 1.15 |
| *Healthy, nutrient-dense core foods (range 0-14)* | 8.0 (2.2) | 11.0 (1.7) | 3.0 (1.8) | 1.1, 4.9 | ***4.1 (5)***** | 1.68 |
| *Total dietary intake (range 0-56)* | 33.7 (6.5) | 46.2 (3.1) | 12.5 (7.6) | 4.5, 20.5 | ***4.0 (5)**** | 1.64 |

*p < 0.05, ** p < 0.01, *** p < 0.001.

**Supplementary Table S5:** Changes in outcomes between baseline and post-program (9-weeks) for children.

| **Outcome** | **Baseline, mean (SD)** | **Post-intervention mean (SD)** | **Mean diff (SD)** | **95%CI** | ***t (df)*** | **Cohen *d*** |
| --- | --- | --- | --- | --- | --- | --- |
| Physical Activity |  |  |  |  |  |  |
| *Days/week meeting PA recommendations* | 2.2 (0.5) | 5.3 (1.4) | 3.2 (1.2) | 1.9, 4.4 | ***6.6 (5)***** | 2.71 |
| Anthropometry |  |  |  |  |  |  |
| *BMI-Z score (kg/m^2^)* | 1.2 (1.6) | 1.3 (1.5) | 0.0 (0.2) | -0.2, 0.2 | *0.2 (6)* | 0.08 |
| Dietary intake |  |  |  |  |  |  |
| *Energy-dense, nutrient poor foods (range 0-42)* | 25.5 (4.3) | 34.7 (3.7) | 9.2 (6.5) | 2.4, 16.0 | ***3.5 (5)**** | 1.41 |
| *Healthy, nutrient-dense core foods (range 0-14)* | 7.5 (2.3) | 11.2 (1.5) | 3.7 (1.9) | 1.7, 5.6 | ***4.8 (5)***** | 1.97 |
| *Total dietary intake (range 0-56)* | 33.0 (6.5) | 45.8 (5.0) | 12.8 (6.5) | 6.1, 19.6 | ***4.9 (5)***** | 1.99 |

*p < 0.05, ** p < 0.01, *** p < 0.001.

**References**

31. S. M. Shepherd, R. H. Delgado, J. Sherwood, et al., “The Impact of Indigenous Cultural Identity and Cultural Engagement on Violent Offending,” BMC Public Health 18 (2018): 1–7.

32. Godin, G. and R. Shephard, *A simple method to assess exercise behavior in the community.* Can J Appl Sport Sci, 1985. **10**(3): p. 141-146.

33. Plotnikoff, R.C., et al., Factors associated with physical activity in Canadian adults with diabetes. Medicine and science in sports and exercise, 2006. 38(8): p. 1526-1534.

34. Lee, S.M., et al., Correlates of children and parents being physically active together. Journal of physical activity and health, 2010. 7(6): p. 776-783.

35. Gwynn, J.D., et al., The reliability and validity of a short FFQ among Australian Aboriginal and Torres Strait Islander and non-Indigenous rural children. Public health nutrition, 2011. 14(3): p. 388-401.

36. Onis, M.d., et al., Development of a WHO growth reference for school-aged children and adolescents. Bulletin of the World health Organization, 2007. 85(9): p. 660-667.

37. Cole, T.J., et al., Establishing a standard definition for child overweight and obesity worldwide: international survey. bmj, 2000. 320(7244): p. 1240.
